# GPR54 Regulates ERK1/2 Activity and Hypothalamic Gene Expression in a Gα_q/11_ and β-Arrestin-Dependent Manner

**DOI:** 10.1371/journal.pone.0012964

**Published:** 2010-09-23

**Authors:** Jacob M. Szereszewski, Macarena Pampillo, Maryse R. Ahow, Stefan Offermanns, Moshmi Bhattacharya, Andy V. Babwah

**Affiliations:** 1 The Children's Health Research Institute, London, Ontario, Canada; 2 Lawson Health Research Institute, London, Ontario, Canada; 3 Department of Obstetrics and Gynaecology, The University of Western Ontario, London, Ontario, Canada; 4 Department of Physiology and Pharmacology, The University of Western Ontario, London, Ontario, Canada; 5 Department of Pharmacology, Max-Planck-Institute for Heart and Lung Research, Bad Nauheim, Germany; University of Oldenburg, Germany

## Abstract

G protein-coupled receptor 54 (GPR54) is a G_q/11_-coupled 7 transmembrane-spanning receptor (7TMR). Activation of GPR54 by kisspeptin (Kp) stimulates PIP_2_ hydrolysis, Ca^2+^ mobilization and ERK1/2 MAPK phosphorylation. Kp and GPR54 are established regulators of the hypothalamic-pituitary-gonadal (HPG) axis and loss-of-function mutations in GPR54 are associated with an absence of puberty and hypogonadotropic hypogonadism, thus defining an important role of the Kp/GPR54 signaling system in reproductive function. Given the tremendous physiological and clinical importance of the Kp/GPR54 signaling system, we explored the contributions of the GPR54-coupled G_q/11_ and β-arrestin pathways on the activation of a major downstream signaling molecule, ERK, using G_q/11_ and β-arrestin knockout mouse embryonic fibroblasts. Our study revealed that GPR54 employs the G_q/11_ and β-arrestin-2 pathways in a co-dependent and temporally overlapping manner to positively regulate ERK activity and pERK nuclear localization. We also show that while β-arrestin-2 potentiates GPR54 signaling to ERK, β-arrestin-1 inhibits it. Our data also revealed that diminished β-arrestin-1 and -2 expression in the GT1-7 GnRH hypothalamic neuronal cell line triggered distinct patterns of gene expression following Kp-10 treatment. Thus, β-arrestin-1 and -2 also regulate distinct downstream responses in gene expression. Finally, we showed that GPR54, when uncoupled from the G_q/11_ pathway, as is the case for several naturally occurring GPR54 mutants associated with hypogonadotropic hypogonadism, continues to regulate gene expression in a G protein-independent manner. These new and exciting findings add significantly to our mechanistic understanding of how this important receptor signals intracellularly in response to kisspeptin stimulation.

## Introduction

G protein-coupled receptor 54 (GPR54) is a G_q/11_coupled 7 transmembrane-spanning receptor (7TMR). Activation of GPR54 by kisspeptin (Kp) stimulates PIP2 hydrolysis, Ca^2+^ mobilization, arachidonic acid release, and ERK1/2 and p38 MAPK phosphorylation [1]. Kp and GPR54 are established regulators of the hypothalamic-pituitary-gonadal axis [2], [3] and loss-of-function mutations in GPR54 are associated with an absence of puberty and hypogonadotropic hypogonadism, a condition characterized by an absence of sexual maturation and low levels of gonadotropic hormones (LH and FSH), in humans. Mice with targeted deletions of GPR54 also have a hypogonadotropic phenotype, confirming the important role of the Kp/GPR54 signaling system in the control of puberty and reproductive function. Given the clinical importance of GPR54, we recently conducted a study that examined the role of GPCR serine/threonine kinase (GRK) 2 and β-arrestin in regulating GPR54 signaling [4]. In that study, we demonstrated that GRK2 stimulates the homologous desensitization of GPR54 and that β-arrestin-2 mediates GPR54 activation of ERK1/2.

Traditionally, β-arrestins are recognized as molecules that mediate the homologous desensitization and clathrin-dependent endocytosis of 7TMRs [5]. However, over the past decade, β-arrestins have been recognized to play much wider roles in biology than previously imagined. Specifically, they serve as molecular scaffolds for signaling proteins that couple 7TMRs to a variety of signaling systems thereby acting as signal transducers in their own right [6], [7]. Among the signaling pathways that β-arrestins couple 7TMRs to, the ERK MAPK represents the prototype for β-arrestin-mediated signaling. Understanding the mechanisms by which β-arrestins activate signaling pathways like ERK1/2 is critical given the important biological responses that lie downstream of these pathways. Such β-arrestin pathway-regulated events include transcription, inflammation, chemotaxis, proliferation and stress fiber formation [6].

Following agonist stimulation, 7TMRs activate MAPK through G protein-dependent mechanisms [8]. The involvement of the G protein-dependent pathway in ERK activation was demonstrated through the use of G protein-dependent pathway inhibitors, such as pertussis toxin, PKC and PKA inhibitors [9]–[11]; G protein pathway-uncoupled receptors and biased agonists [12]–[15]. For several 7TMRs, including the G_q/11_coupled proteinase-activated receptor 2 (PAR2) and angiotensin type 1A receptor (AT1AR) [12], [16]-[17], the Gs-coupled β2 adrenergic receptor (β2AR) [13] the G_q/11_, Gs-coupled type I PTH/PTH-related peptide receptor (PTH1R) [10] and the Gi-coupled dopamine D2 receptor (D2R), G protein activation of the MAPK signal is rapid and detected within 2-5 minutes following agonist treatment. As clearly shown for the AT1AR, β2AR and PTH1R, G protein activation of ERK is also transient peaking within 2-10 minutes of agonist treatment [9]–[10], [13], [17] presumably due to the subsequent β-arrestin-mediated desensitization of the receptor.

In a distinct but related process to the G protein-dependent activation of ERK, β-arrestins scaffold the MAPK signaling molecules, Raf-1, MEK1 and ERK, thereby mediating the phosphorylation and activation of ERK 1/2 [18]. The involvement of the β-arrestin-dependent pathway in ERK activation was demonstrated through the use of β-arrestin siRNAs [9], [10], [12], [13] and MEFs derived from β-arrestin null mice [19]. For some receptors, such as PAR2, β2AR and PTH1R, both β-arrestin-1 and -2 potentiate signaling to ERK [10], [13], [16], however, consistent with their varied roles in regulating receptor desensitization and internalization, β-arrestin-1 inhibits while β-arrestin-2 stimulates (reciprocal effect) ERK activation downstream of the AT1AR [9].

Generally, G protein and β-arrestin-dependent ERK activation can be resolved into two temporal phases; an early G protein-dependent phase that is rapid and transient and a late β-arrestin-dependent phase that is slower and more persistent, as shown for the AT1AR, β2AR, vasopressin 2 receptor (V2R) and PTH1R [9], [10], [13], [20]. For these receptors, including PAR2, it has also been shown that the G protein and β-arrestin-dependent pathways are independent of each other and ERK activation could still be observed if one of the pathways was blocked [9]–[10], [13], [16], [20]–[21]. While many examples of β-arrestin-dependent signaling are G protein-independent, some studies, such as that by Barnes et al. [22], report that the AT1AR-dependent robust activation of RhoA requires the cooperation of both the G protein and β-arrestin-1-dependent pathways.

For several receptors it has been determined that activated ERK translocates from the cytosol to the nucleus where it regulates gene transcription. With the discovery that ERK can be activated via the G protein and β-arrestin-dependent pathways, studies have revealed that the eventual cellular location of activated ERK is linked to the pathway mediating its activation. In cells expressing PAR2 and AT1AR, ERK activated via G protein-dependent pathway were shown to translocate to the nucleus, whereas β-arrestin-activated ERK remained in the cytosol [6]. This pathway-dependent cellular location requires further investigation since it was demonstrated that β-arrestin-2 inhibited nuclear translocation of ERK following AT1AR activation [21] but enhanced nuclear translocation of ERK following β2AR activation [23]. These two apparently contrasting studies suggest that the eventual cellular location of the activated ERK is at least in part a receptor-specific event.

Having established the role of β-arrestin-2 in coupling GPR54 to ERK [4] and given the biological importance of understanding the mechanism by which GPR54 activates ERK, we set out to conduct a detailed mechanistic analysis on the roles of the G_q/11_ and β-arrestin-1- and -2-dependent pathways in regulating ERK activation and gene expression following GPR54 activation. These studies were conducted in mouse embryonic fibroblast cell lines bearing G_q/11_ and β-arrestin-1 and -2 gene deletions or disruptions and a mouse GnRH hypothalamic cell line expressing reduced levels of β-arrestin-1 and -2. Our results reveal that β-arrestin-1 inhibits while β-arrestin-2 and G_q/11_ activate ERK1/2 in a co-dependent manner following GPR54 activation. Our results also reveal that β-arrestin-1 and -2 regulate distinct downstream responses in gene expression.

## Results

### GPR54 is coupled to ERK MAPK pathway in WT MEFs

**Figure 1:**
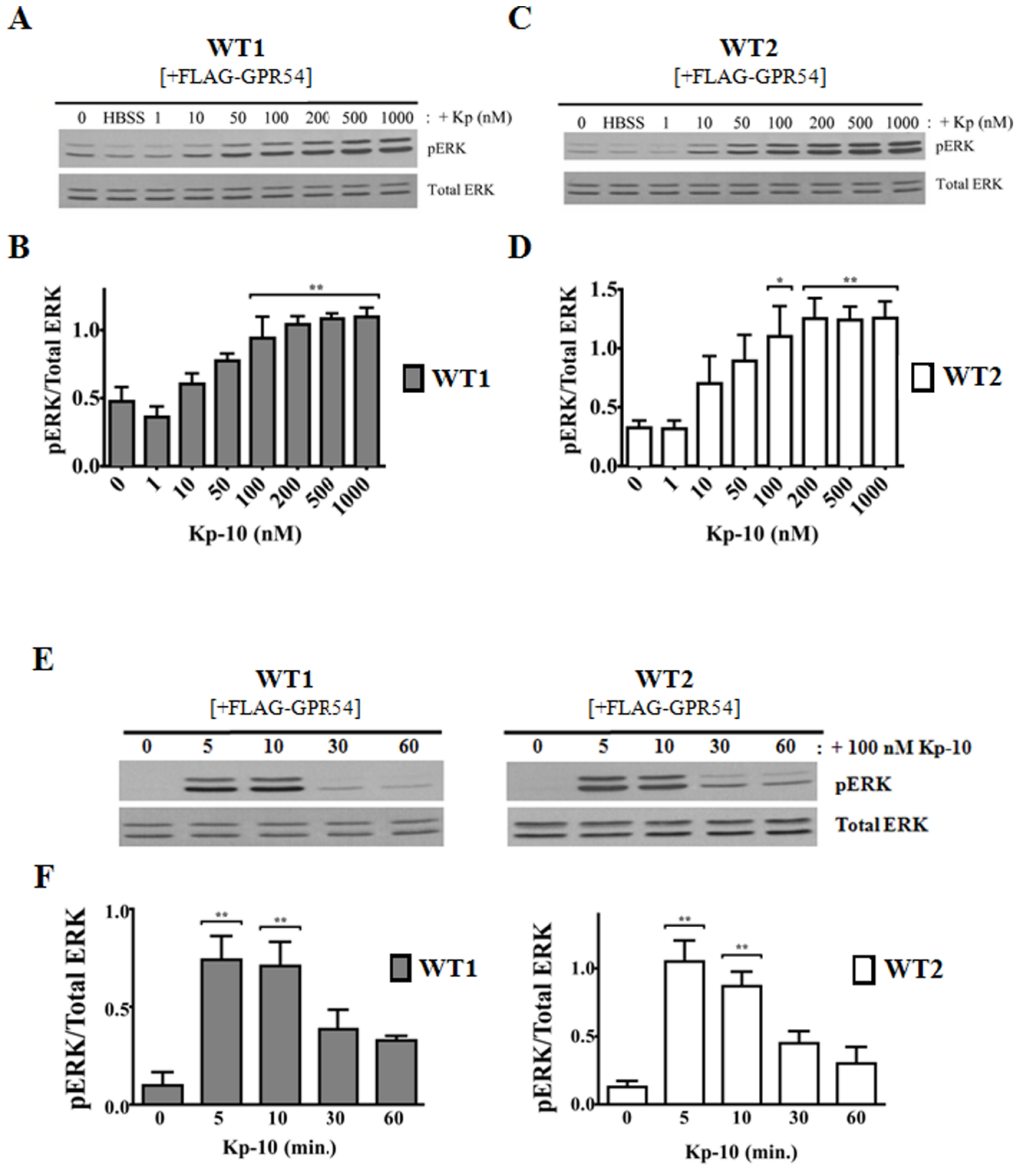
GPR54 is coupled to ERK MAPK pathway in WT MEFs. Representative autoradiographs (A and C) and densitometric analyses (B and D) showing the expression of total and activated ERK1/2 in GPR54 overexpressing WT1 (β-arrestin-1 KO parent) and WT2 (β-arrestin-2 and 1/2 KO parent) MEFs following 10-minute treatment with increasing concentrations of Kp-10 (0–1000 nM). Representative autoradiograph (E) and densitometric analysis (F) showing the expression of total and activated ERK1/2 in GPR54 overexpressing WT1 and WT2 parental MEF cell lines following 100 nM Kp-10 treatment (for the indicated time points: 0, 5, 10, 30 and 60 minutes). Western blot analyses were done using monoclonal anti-ERK1/2 and anti-phospho ERK1/2 antibodies. The data represent the mean ± S. E. of 4 independent experiments. *P<0.05; **P<0.01vs control (0 min.).

Using the β-arrestin-1 and -2 WT parental MEF cell lines expressing exogenous GPR54, we determined whether there was a functional GPR54/ERK signaling system. This was done by treating cells with increasing concentrations of Kp-10 (1 to 1000 nM) for 10 minutes following which ERK activation was assessed by western blotting. Ten minutes were chosen as the treatment time since several studies conducted in a variety of GPR54 expressing cell lines showed ERK remains highly elevated at 10 minutes [1], [24]. Our results showed that relative to HBSS (vehicle) treated and unstimulated cells, ERK activation was visually detectable in both WT lines following 10 nM Kp-10 stimulation (Fig. 1A–D). As Kp-10 concentration increased so did pERK levels with activation leveling off at 100 nM Kp-10 (Fig. 1A–D). Next, we conducted a time-course study in which GPR54 overexpressing β-arrestin-1 and -2 WT parental MEF cell lines were treated with 100 nM Kp-10 for 5, 10, 30 and 60 minutes following which ERK activation was assessed by western blotting. Our results revealed that in both WT parental MEFs, ERK was activated rapidly (within 5 minutes of Kp-10 treatment), remained stable for up to at least 10 minutes and decreased thereafter (Fig. 1E, F).

### GPR54 activates ERK1/2 in a β-arrestin-dependent manner

**Figure 2:**
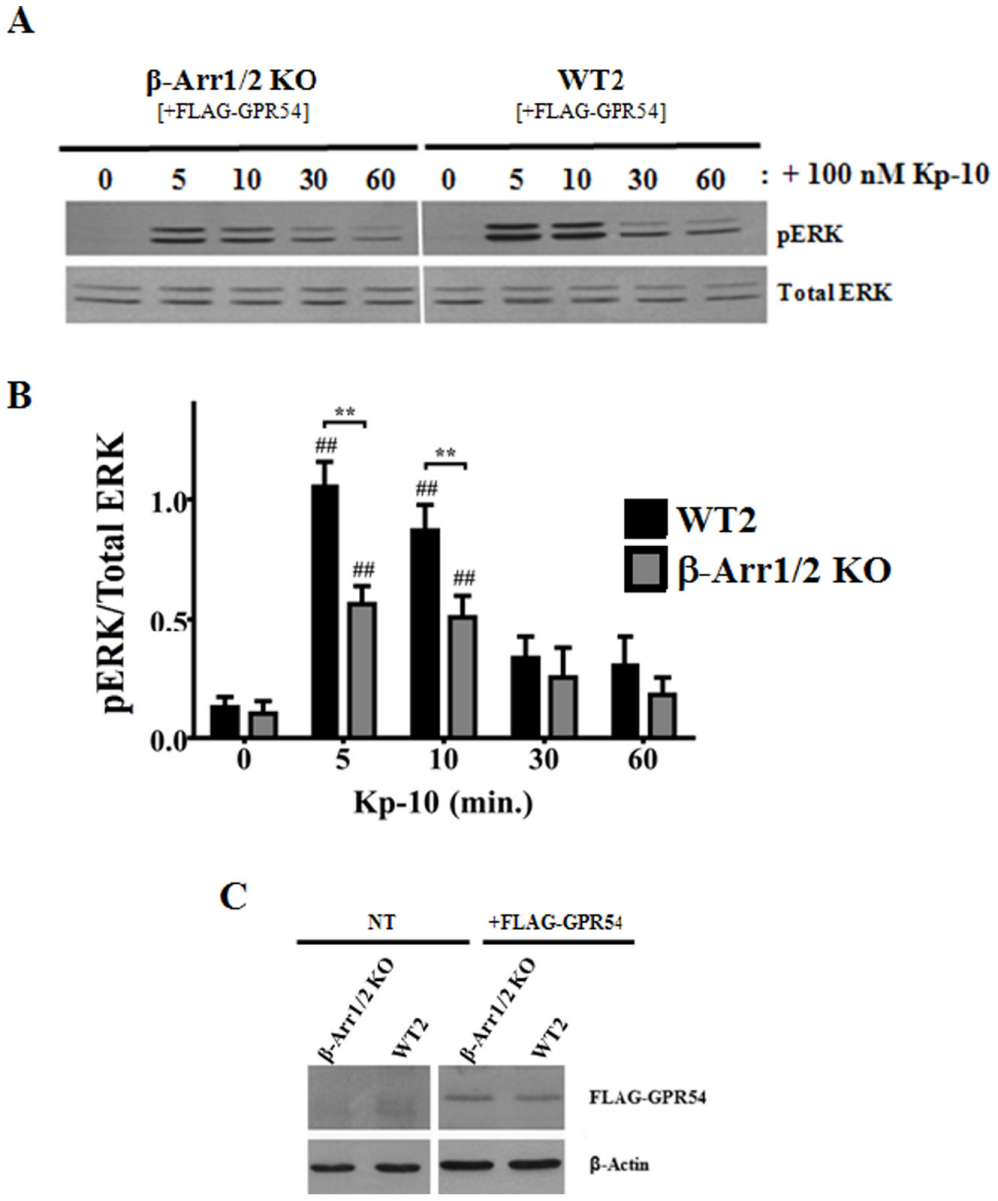
GPR54 activates ERK1/2 in a β-arrestin-dependent manner. Representative autoradiograph (A) and densitometric analysis (B) showing the expression of total and activated ERK1/2 in GPR54 overexpressing β-Arr1/2 double KO (β-Arr1/2 KO) and corresponding wild type parental (WT2) MEFs following stimulation with 100 nM Kp-10 (for the indicated time points). (C) Representative Western blot confirming absence and presence of expression of FLAG-GPR54 in non-electroporated (NT) and FLAG-GPR54 overexpressing β-Arr1/2 KO and WT2 MEF cells, respectively. Western blot analyses were done using monoclonal anti-ERK1/2, anti-phospho ERK1/2, and anti-DDK (FLAG) antibodies. The data represent the mean ± S. E. of 4 independent experiments. ^##^ *P*<0.01 vs 0 min. control (within the specific cell line). **P<0.01 vs respective wild-type control at the indicated time point.

To begin assessing the effect of β-arrestin-1 and -2 in regulating the GPR54-dependent activation of ERK in MEFs, we began by determining whether there was any detectable change in pERK levels in the β-arrestin-1/2 double knockout (β-arr1/2 KO) MEFs relative to its WT parental MEFs (WT2). GPR54 overexpressing β-arrestin-1/2 KO MEFs and its WT parent were treated with Kp-10 (100 nM) for 5, 10, 30 and 60 minutes following which pERK1/2 levels were assessed by western blotting. Our results revealed that a loss of β-arrestin-1/2 expression led to significantly reduced (*P*<0.01) pERK levels relative to its WT parent at 5 and 10 minutes following Kp-10 treatment (Fig. 2A, B). To ensure that the differences we were observing in ERK activation were not due to differences in FLAG-GPR54 expression levels between the KO cells and their WT parental control cells, we routinely western blotted for FLAG-GPR54 expression using an anti-DDK (FLAG) monoclonal antibody. Our data showed that receptor expression was similar between the KO and control cell lines (Fig. 2C). Finally, although the levels of pERK1/2 we observed in the GPR54 overexpressing β-arrestin-1/2 KO MEFs were reduced at 5 and 10 minutes compared to their wild-type parental MEFs, there is still a significant increase (*P*<0.01) above basal in ERK1/2 activation in the β-arrestin-1/2 KO cell line at 5 and 10 minutes Kp-10 treatment (Fig. 2A, B).

### GPR54 stimulation of ERK1/2 is enhanced in the absence of β-arrestin-1

**Figure 3:**
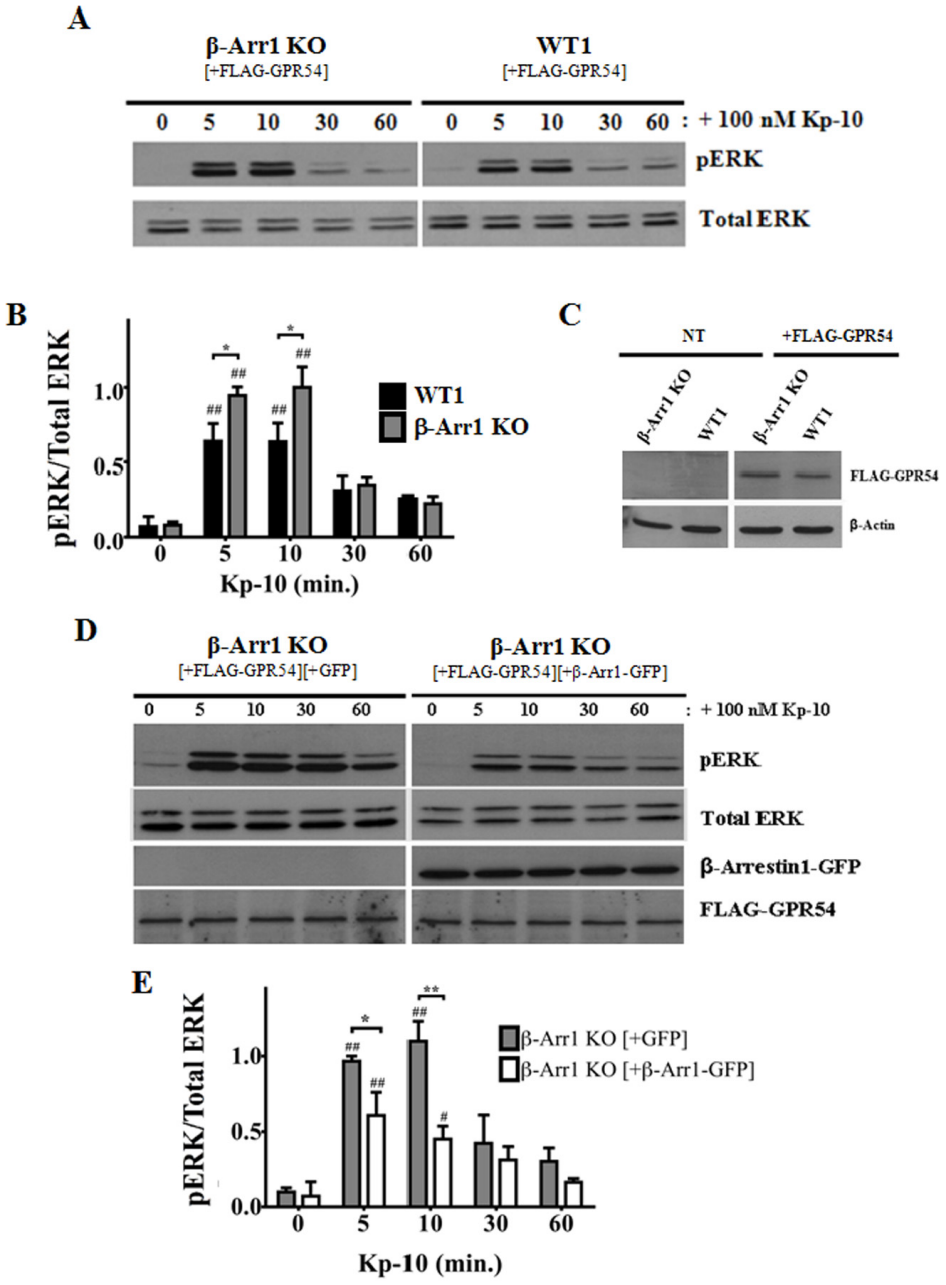
GPR54 stimulation of ERK1/2 is enhanced in the absence of β-arrestin-1. Representative autoradiograph (A) and densitometric analysis (B) showing the expression of total and activated ERK1/2 in GPR54 overexpressing β-Arr1 KO and corresponding wild type parental (WT1) MEFs following stimulation with 100 nM Kp-10 (for the indicated time points). (C) Representative western blot confirming absence and presence of expression of FLAG-GPR54 in non-electroporated (NT) and FLAG-GPR54 overexpressing β-Arr1 KO and WT1 MEFs, respectively. Representative autoradiograph (D) and densitometric analysis (E) showing the expression of total and activated ERK1/2 following stimulation with 100 nM Kp-10 (for indicated time points) of FLAG-GPR54 overexpressing β-Arr1 KO MEFs co-transfected with either GFP vector (grey bars) or β-Arr1-GFP (white bars). All western blot analyses were done using monoclonal anti-ERK1/2 and anti-phospho ERK1/2 antibodies. Monoclonal anti-β-Arr1 and anti-DDK (FLAG) antibodies were also used. The data represent the mean ± S. E. of 4 independent experiments (B) or the mean ± S. E. of 3 independent experiments (E). ^#^ *P*<0.05, ^##^ *P*<0.01 vs 0 min. control (within the specific cell line). *P<0.05, **P<0.01 vs respective wild-type (or ‘add-back’) control at the indicated time point.

Since it is established that β-arrestin-1 and -2 exert multiple effects on ERK activation [18], we next assessed the relative contribution of each isoform on GPR54-dependent ERK activation beginning with β-arrestin-1. Here we observed that following Kp-10 treatment, the pERK levels in both the GPR54 overexpressing β-arrestin-1 KO and WT parental MEFs (WT1) increased above basal at 5 (*P*<0.01) and 10 minutes (*P*<0.01) Kp-10 treatment. Interestingly, relative to its WT parental MEFs, pERK levels were also significantly higher (*P*<0.05) at 5 and 10 minutes following Kp-10 treatment (Fig. 3A, B). At 30 and 60 minutes, however, pERK levels were the same between the β-arrestin-1 KO and WT parental MEFs. As we had demonstrated before, here we also determined and demonstrated by western blotting that the differences we were observing in ERK activation were not due to differences in FLAG-GPR54 expression levels between the KO cells and their WT parental control cells (Fig. 3C). To confirm the observed differential ERK activation, we then conducted a β-arrestin-1 ‘add-back’ experiment. Here, GPR54 overexpressing β-arrestin-1 KO MEFs, co-transfected with either GFP vector (grey bars) or β-arrestin1-GFP (white bars), were stimulated with 100 nM Kp-10 and then assessed for ERK1/2 activation, FLAG-GPR54, and β-arrestin1-GFP expression by western blotting (Fig. 3D). Consistent with the above findings, we observed that co-expression of FLAG-GPR54 with β-arrestin1-GFP resulted in a significant decrease in pERK1/2 levels following 5 (*P*<0.05) and 10 minutes (*P*<0.01) Kp-10 treatment, relative to β-arrestin-1 KO MEFs co-expressing FLAG-GPR54 and GFP vector (Fig. 3E)

### GPR54 positively regulates ERK1/2 activation in a β-arrestin-2-dependent manner

**Figure 4:**
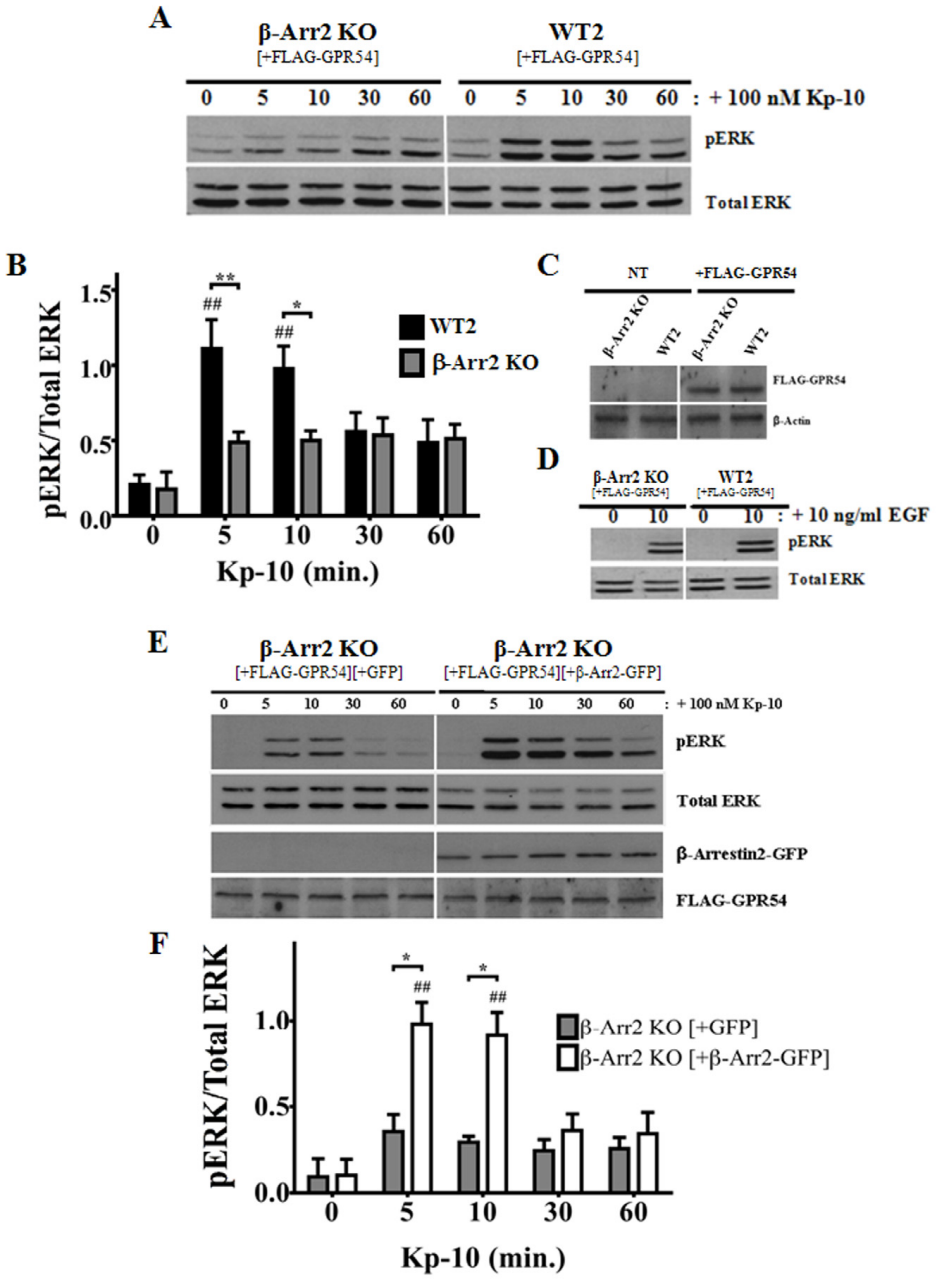
GPR54 positively regulates ERK1/2 activation in a β-arrestin-2-dependent manner. Representative autoradiograph (A) and densitometric analysis (B) showing the expression of total and activated ERK1/2 in GPR54 overexpressing β-Arr2 KO and corresponding wild type parental (WT2) MEFs following stimulation with 100 nM Kp-10 (for the indicated time points). (C) Representative Western blot confirming absence and presence of expression of FLAG-GPR54 in non-electroporated (NT) and FLAG-GPR54 overexpressing β-Arr2 KO and WT2 MEFs, respectively. (D) Representative western blot showing the expression of total and activated ERK 1/2 following treatment of the β-Arr2 KO and corresponding wild type parental (WT2) MEFs with 10 ng/ml EGF (for the indicated time point). This was used as a control to assess whether the ERK1/2 pathway was still functional in these cells. Representative autoradiograph (E) and densitometric analysis (F) showing the expression of total and activated ERK1/2 following stimulation with 100 nM Kp-10 (for indicated time points) of FLAG-GPR54 overexpressing β-Arr2 KO MEFs co-transfected with either GFP vector (grey bars) or β-Arr2-GFP (white bars). All western blot analyses were done using monoclonal anti-ERK1/2 and anti-phospho ERK1/2 antibodies. Monoclonal anti-β-Arr2 and anti-DDK (FLAG) antibodies were also used. The data represent the mean ± S. E. of 4 independent experiments (B) or the mean ± S. E. of 3 independent experiments (F). ^##^ *P*<0.01 vs 0 min. control (within the specific cell line). *P<0.05, ** *P*<0.01vs respective wild-type (or ‘add-back’) control at the indicated time point.

Next, we examined the relative contribution of β-arrestin-2 in GPR54 activation of ERK1/2. The data showed that visually, pERK levels in the β-arrestin-2 KO MEFs increased only slightly above basal following Kp-10 treatment and this was in marked contrast to the WT parental cells (Fig. 4A, B). However, when quantified, this was not significant. Next, we again determined and demonstrated by western blotting that the differences we were observing in ERK activation were not due to differences in FLAG-GPR54 expression levels between the KO cells and their WT parental control cells (Fig. 4C). Additionally, to ensure that the inability of the β-arrestin-2 KO line to stimulate a significant increase in pERK following Kp-10 treatment was not due to a general defect in ERK activation by this KO line, we demonstrated that EGF (10 ng/ml) was able stimulate robust ERK activation following 10 minutes of stimulation while Kp-10 was unable to at that corresponding time point (Fig. 4D vs. 4A). We also conducted a β-arrestin-2 ‘add-back’ experiment to confirm our results. Here, GPR54 overexpressing β-arrestin-2 KO MEFs, co-transfected with either GFP vector (grey bars) or β-arrestin2-GFP (white bars), were stimulated with 100 nM Kp-10 and then assessed for ERK1/2 activation, FLAG-GPR54, and β-arrestin2-GFP expression by western blotting (Fig. 4E). Consistent with the above findings, we observed that co-expression of FLAG-GPR54 with β-arrestin2-GFP resulted in a significant increase (*P*<0.05) in pERK1/2 levels at 5 and 10 minutes Kp-10 treatment, relative to β-arrestin-2 KO MEFs co-expressing FLAG-GPR54 and GFP vector (Fig. 4F).

### GPR54 positively regulates ERK1/2 activation in a G_q/11_-dependent manner

**Figure 5:**
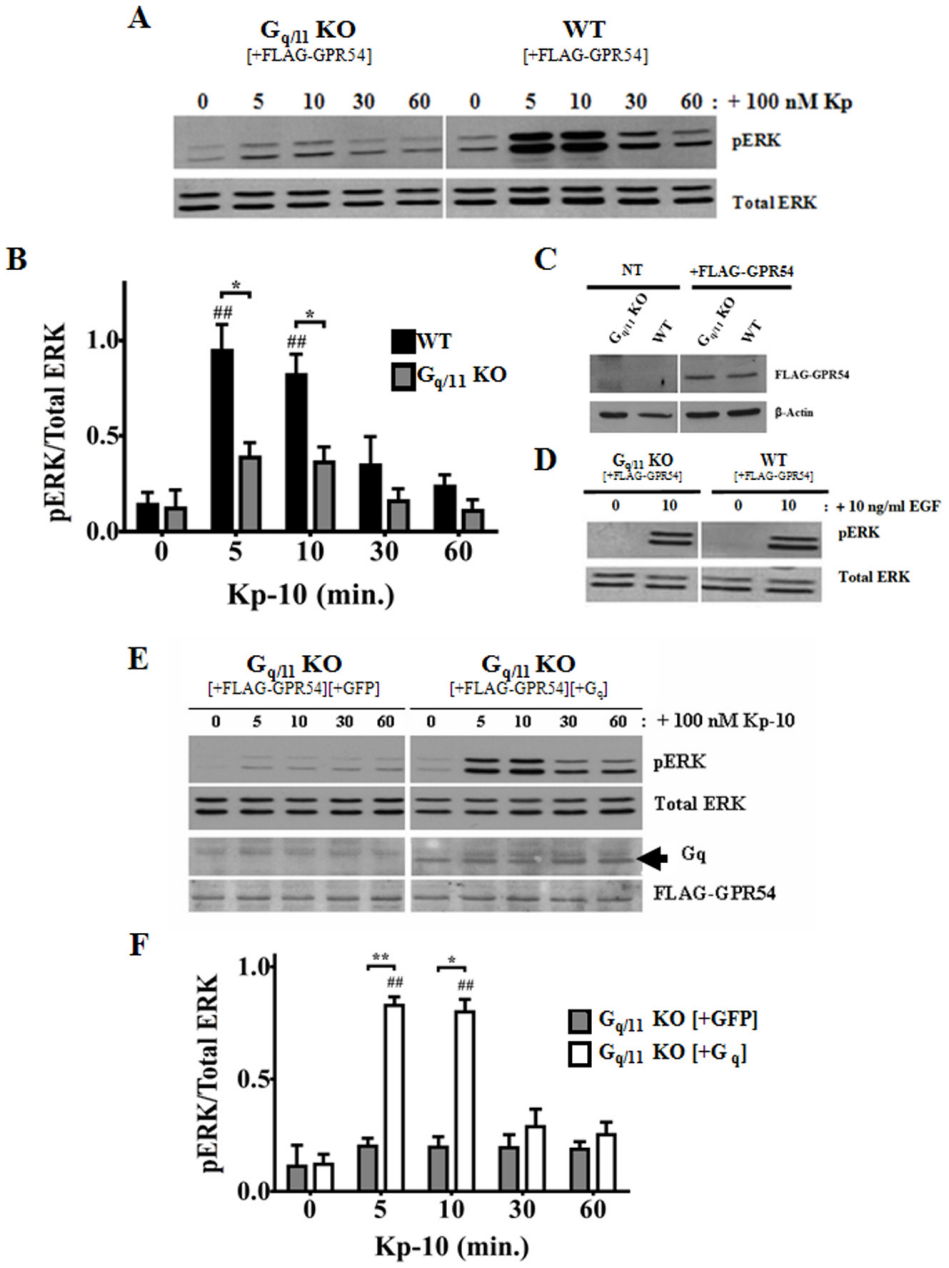
GPR54 positively regulates ERK1/2 activation in a G_q/11_-dependent manner. Representative autoradiograph (A) and densitometric analysis (B) showing the expression of total and activated ERK1/2 in GPR54 overexpressing G_q/11_ KO and corresponding WT parental MEFs following stimulation with 100 nM Kp-10 (for the indicated time points). (C) Representative Western blot confirming absence and presence of expression of FLAG-GPR54 in non-electroporated (NT) and FLAG-GPR54 overexpressing G_q/11_ KO and WT MEFs, respectively. (D) Representative western blot showing the expression of total and activated ERK 1/2 following treatment of the G_q/11_ KO and corresponding WT parental MEFs with 10 ng/ml EGF (for the indicated time points). This was used as a control to assess whether the ERK1/2 pathway was still functional in these cells. Representative autoradiograph (E) and densitometric analysis (F) showing the expression of total and activated ERK1/2 following stimulation with 100 nM Kp-10 (for indicated time points) of GPR54 overexpressing G_q/11_ KO MEFs co-transfected with either GFP vector (grey bars) or untagged G_q_ (white bars). All western blot analyses were done using monoclonal anti-ERK1/2 and anti-phospho ERK1/2 antibodies. Polyclonal anti-G_q_ and monoclonal anti-DDK (FLAG) antibodies were also used. The data represent the mean ± S. E. of 4 independent experiments (B) or the mean ± S. E. of 3 independent experiments (F). ^##^ *P*<0.01 vs 0 min. control (within the specific cell line). *P<0.05, ** *P*<0.01 vs respective wild-type (or ‘add-back’) control at the indicated time point.

Since G_q/11_coupled receptors activate ERK robustly via the G_q/11_ pathway, we determined the role of the G_q/11_coupled pathway in the GPR54-dependent activation of ERK. GPR54 overexpressing G_q/11_ KO MEFs and its WT parent were treated with Kp-10 (100 nM) for 5, 10, 30 and 60 minutes following which pERK1/2 levels were assessed by western blotting. The results were very similar to that observed for the β-arrestin-2 KO MEFs. That is, visually, pERK levels in the G_q/11_ KO MEFs increased only slightly above basal following Kp-10 treatment and this was in marked contrast to the WT parental cells (Fig. 5A, B). Again, when quantified, this was not significant. Next, by western blotting, we confirmed that differences we were observing in ERK activation between the KO cells and their WT parental control cells were not due to differences in FLAG-GPR54 expression levels (Fig. 5C). To confirm that the inability of the G_q/11_ KO MEFs to trigger a strong response in ERK activation following Kp-10 treatment was not due to a general defect in ERK activation in this KO line, we demonstrated that 10 minutes of EGF treatment was able stimulate robust ERK activation while Kp-10 was unable to at that corresponding time point (Fig. 5D vs. 5A). Once again, we conducted a Gq ‘add-back’ experiment to confirm our results. Here, GPR54 overexpressing G_q/11_ KO MEFs, co-transfected with either GFP vector (grey bars) or Gq (white bars), were stimulated with 100 nM Kp-10 and then assessed for ERK1/2 activation, FLAG-GPR54 and Gq expression by Western blotting. Consistent with the above findings, we observed that co-expression of FLAG-GPR54 with exogenous Gq resulted in a significant increase (*P*<0.01) in pERK1/2 levels at 5 and 10 minutes Kp-10 treatment, relative to G_q/11_ KO MEFs co-expressing FLAG-GPR54 and GFP vector (Fig. 5F).

### β-arrestin-2 and G_q/11_ regulate the GPR54-dependent ERK1/2 activation in a very rapid and temporally overlapping manner

**Figure 6:**
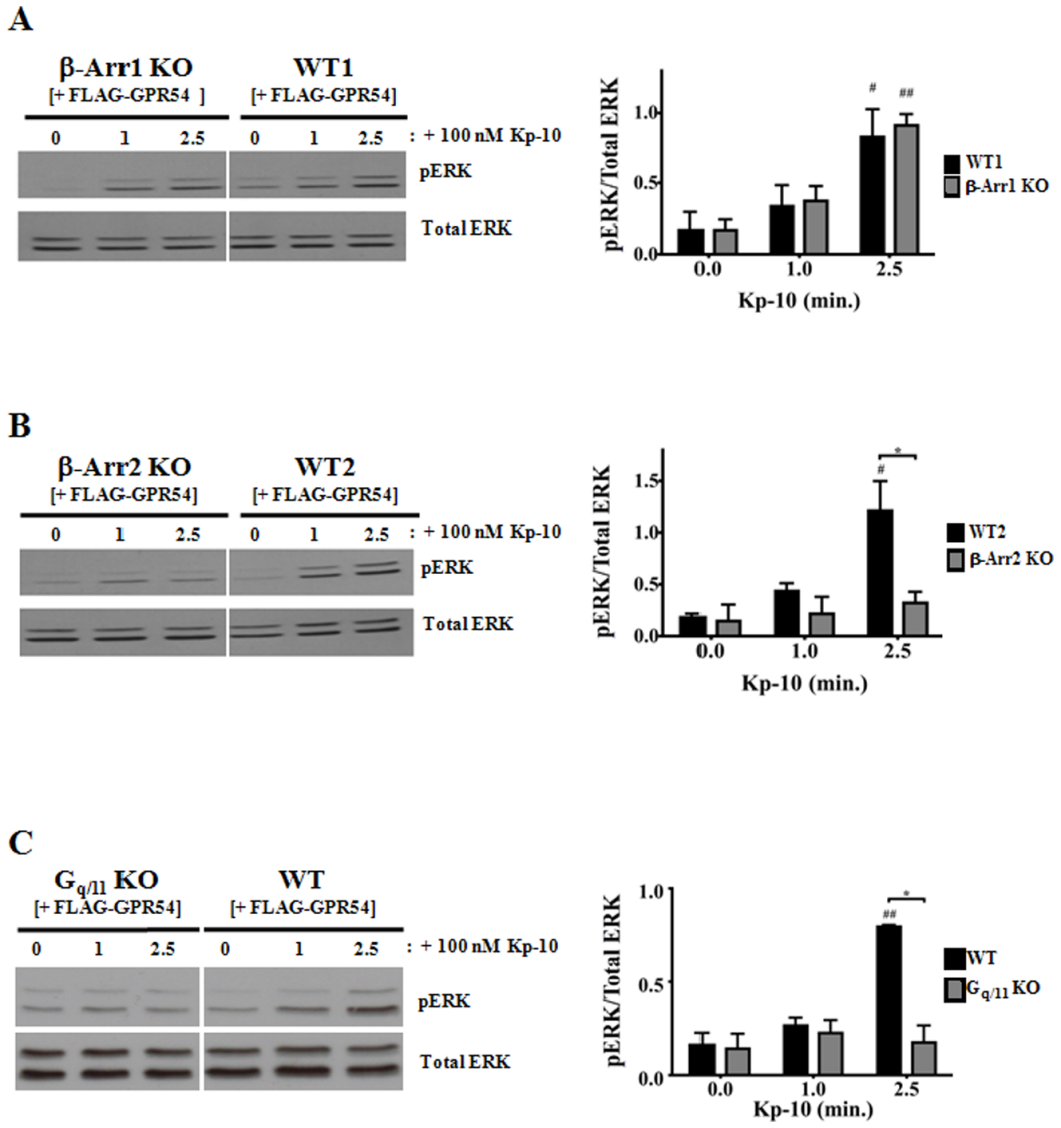
β-arrestin-2 and G_q/11_ regulate the GPR54-dependent ERK1/2 activation in a rapid and temporally overlapping manner. Representative autoradiographs and densitometric analyses showing the expression of total and activated ERK1/2 in GPR54 overexpressing (A) β-Arr1 KO and WT1 MEFs; (B) β-Arr2 KO and WT2 MEFs; and (C) G_q/11_ KO and WT MEFs following stimulation with 100 nM Kp-10 (for the indicated time points). All western blot analyses were done using monoclonal anti-ERK1/2 and anti-phospho ERK1/2 antibodies. The data represent the mean ± S. E. of 3 independent experiments. # *P*<0.05, ##*P*<0.01 vs 0 min. control (within the specific cell line). *P<0.05 vs respective wild-type control at the indicated time point.

Thus far, our analyses examined GPR54-dependent ERK activation following 5, 10, 30 and 60 minutes of Kp-10 treatment. However, since it was demonstrated for the AT1AR that G protein activation of ERK peaks as early as two minutes following AngII treatment (9), we assessed the regulatory effect of G_q/11_ and β-arrestins on very early ERK activation following 1 and 2.5 minutes of Kp-10 treatment. The data revealed that as early as 2.5 minutes following Kp-10 treatment, significantly reduced pERK levels were detected in the β-arrestin-2 (*P*<0.05) and G_q/11_ (*P*<0.05) KO cells compared to their corresponding wild-type parental cell lines (Fig. 6B, C). At these early time points, β-arrestin-1 did not have an effect on ERK activation following Kp-10 treatment (Fig. 6A). Thus it appears that β-arrestin-2 and G_q/11_ regulate the GPR54-dependent activation of ERK in a very rapid and temporally overlapping manner.

### G_q/11_ and β-arrestin-dependent GPR54-coupled pathways regulate the spatial distribution of pERK

**Figure 7:**
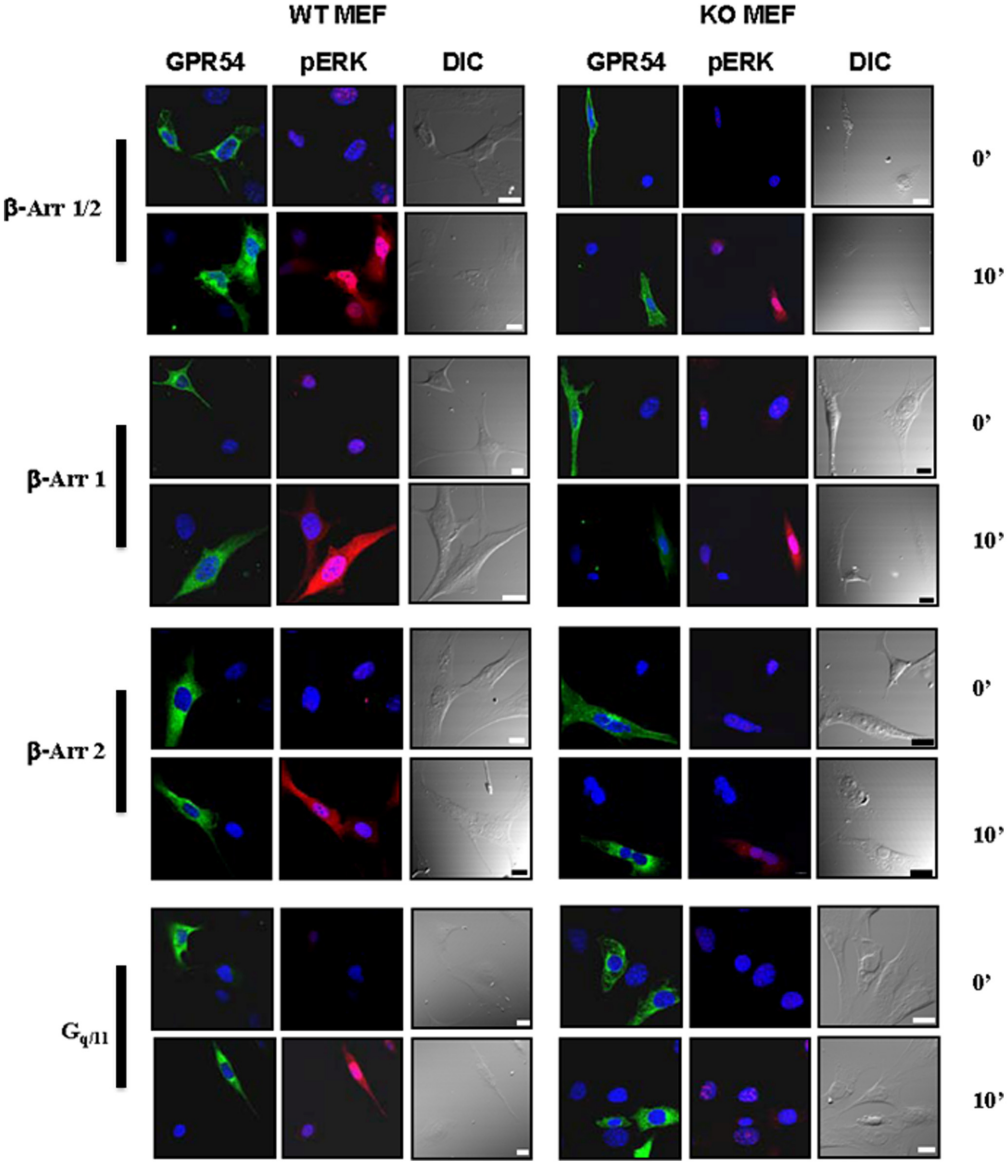
G_q/11_ and β-arrestin-dependent GPR54-coupled pathways regulate the spatial distribution of pERK. Immunofluorescence analysis of GPR54 expression and pERK levels in GPR54-EYFP overexpressing G_q/11_ and β-Arr KO MEFs and their WT parents before and after 10 minutes of Kp-10 treatment. Each inset contains cells that express and do not express GPR54 (green; first and fourth columns). These cells were analyzed for pERK levels (red fluorescence) before (0 minutes) and after (10 minutes) Kp-10 treatment. Differential interference contrast (DIC) images of cells and DAPI-stained nuclei (with GPR54 and pERK) are shown to provide greater spatial information. In all cases, the same image acquisition settings were applied, thus receptor and pERK levels can be compared between cell lines and treatments. The images are representative of 3 independent experiments.

While immunoblotting of pERK following Kp-10 treatment provides powerful data on pERK cellular levels it does not provide any information on the spatial distribution of pERK within the cell. Therefore, to complement the earlier studies, we conducted a spatial analysis of pERK levels in the GPR54 overexpressing G_q/11_ and β-arrestin KO MEFs and their WT parents before and after Kp-10 treatment. To do this, we randomly selected a field of view that had a receptor (GPR54-YFP) expressing and non-expressing cell. The non-expressing cell served as the control cell. It must be noted that during the acquisition of image data, the same image acquisition settings were applied, thus receptor and pERK levels can be compared between the cell lines and treatments. Visual analysis of pERK levels yielded data that were in general agreement with the immunoblot data. The data first revealed that in the absence of Kp-10 treatment, pERK was barely detectable in any cell, regardless of whether they expressed GPR54 (Fig. 7). However, following 10 minutes of treatment of all WT MEFs expressing GPR54, pERK levels increased dramatically and it was strongly distributed in both the cytosol and nucleus (Fig. 7). In the β-arrestin-1/2 KO and -1 KO, following treatment, pERK was also well expressed throughout the cell (Fig. 7). However, in both the β-arrestin-2 KO and G_q/11_ KO cells, pERK levels were only slightly higher than that observed in untreated cells (Fig. 7).

### L148S interacts with β-arrestin-1 and 2 while GPR54 continues to modulate gene expression in the absence of G_q/11_

**Figure 8:**
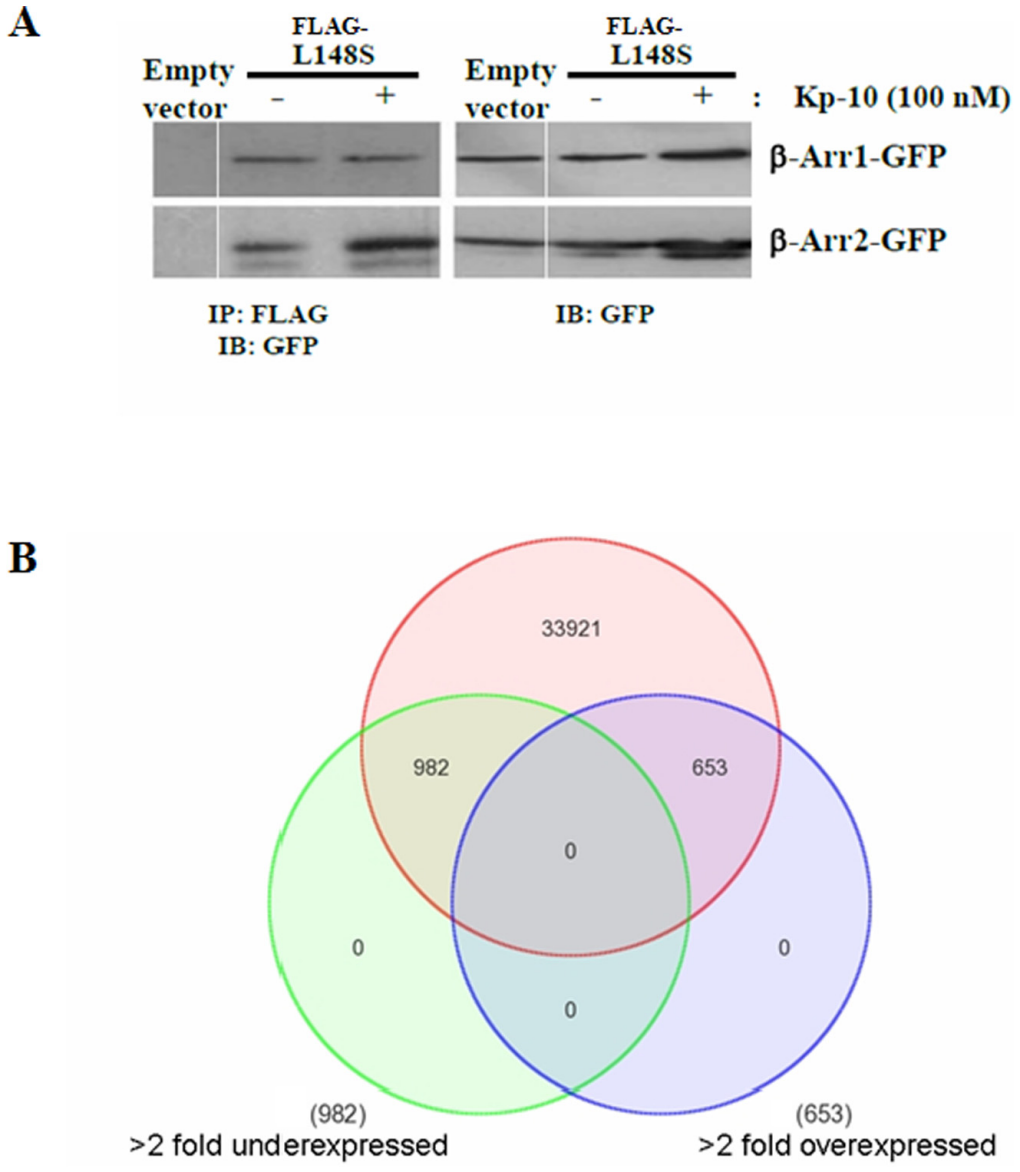
L148S interacts with β-arrestin-1 and 2 while GPR54 continues to modulate gene expression in the absence of G_q/11_. (A) Representative autoradiograph showing the co-immunoprecipitation of β-arrestin-1 and β-arrestin-2 with FLAG-L148S. HEK 293 cells were transfected with 10 µg of β-arrestin-1 or -2-GFP and 10 µg of FLAG-L148S or empty FLAG vector. Cells were left untreated or stimulated with 100 nM Kp-10 for 5 minutes. Lysates were prepared, immunoprecipitated with mouse anti-FLAG antibody, and immunoblotted with mouse anti-GFP antibody. The expression of β-arrestin-1 or -2-GFP in 50 µg of total protein from the corresponding HEK 293 cell lysates is also shown. The data represent the mean ± SE for three independent experiments. IB: Immunoblotting; IP: immunoprecipitation. (B) Gene microarray analysis of G_q/11_ KO MEFs versus WT control MEFs that were treated with Kp-10 for six hours. The data is presented as a three-way Venn diagram and shows the overlap of genes changing greater than 2 fold in their expression (up or down) as a proportion of all the probesets on the Affymetrix Mouse Gene 1.0 ST Array. In total, 1,635 out of a total of 35,556 probesets are altered greater than 2-fold (4.6%) (*P*<0.05).

Since our data clearly showed that GPR54 had the capacity to display G protein-independent signaling by coupling to the β-arrestin pathway, we tested whether a naturally occurring GPR54 mutant, L148S, which was demonstrated to be functionally uncoupled from G_q/11_ [25] would interact with β-arrestin-1 and 2. Through the use of a co-immunoprecipitation assay, we demonstrated that it did and that the interaction was also very robust (Fig. 8A) and identical to what we previously observed for the interaction between WT GPR54 and β-arrestin-1 and 2 [4]. Next, we reasoned that if L148S could interact with β-arrestin-1 and 2, it might mean that GPR54 molecules could regulate downstream signaling events like gene expression in the absence of G_q/11_. To test this, G_q/11_ KO MEFs expressing FLAG-GPR54 were serum-starved overnight and then treated with Kp-10 for 6 hours in serum-free media. Total RNA was extracted and used to conduct a microarray analysis of gene expression using the Affymetrix Mouse Gene 1.0 ST gene array. Our data revealed that relative to control MEFs, the G_q/11_ KO MEFs had a clear and significant (*P*<0.05) effect on gene expression following Kp-10 treatment (Fig. 8B). In total, 1,635 (4.6%) out of a total of 35,556 probesets were altered greater than 2-fold (up or down).

### GPR54-mediated gene expression in the hypothalamic cell line, GT1-7, is altered following reduced β-arrestin-1 and 2 expression

**Figure 9:**
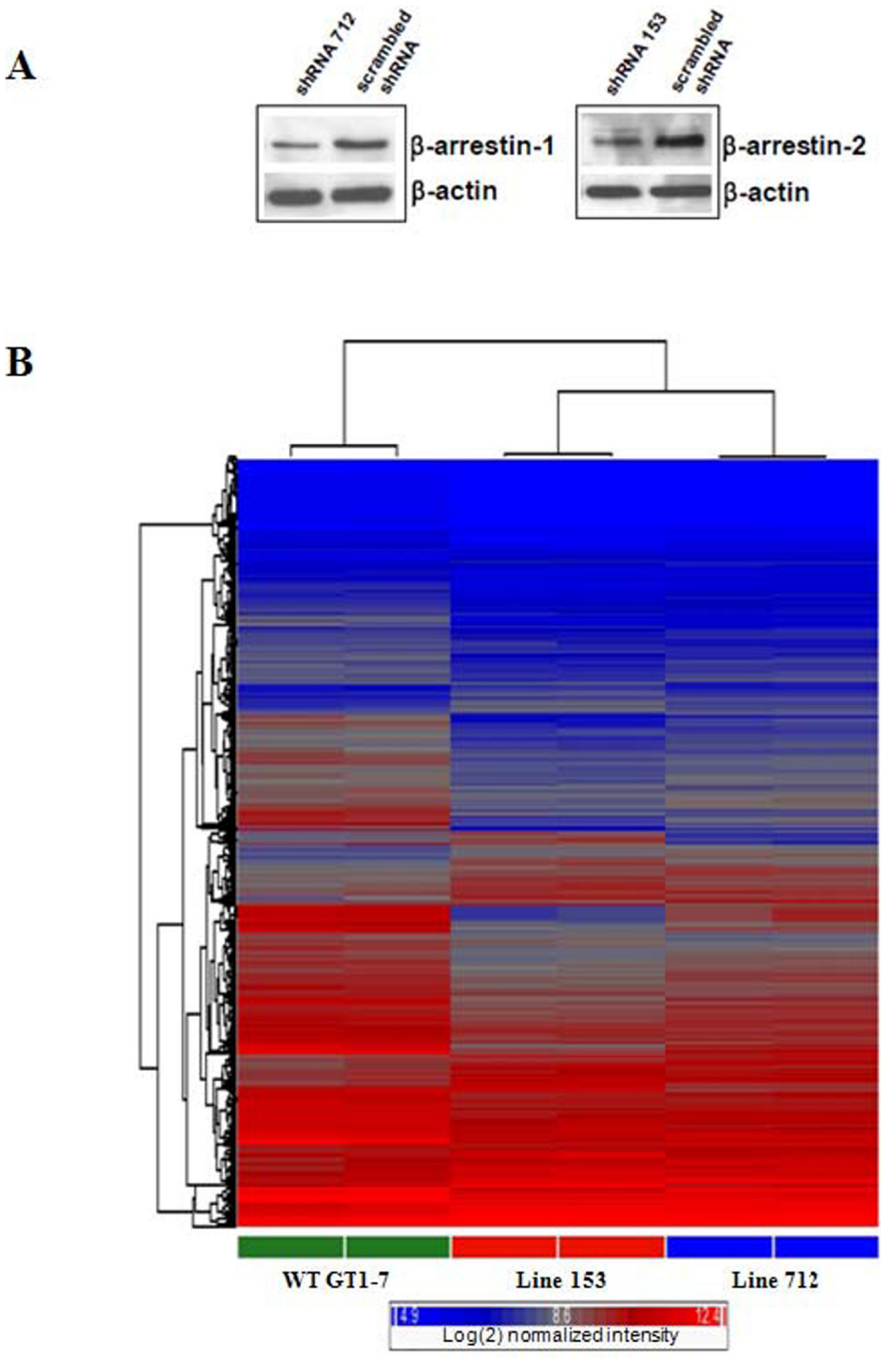
GPR54-mediated gene expression in the hypothalamic cell line, GT1-7 is altered following reduced β-arrestin-1 and 2 expression. (A) GT1-7 cell lines stably expressing shRNAs against β-arrestin-1 (line 712) and β-arrestin-2 (line 153) were created and β-arrestin expression was determined by western blotting. This data revealed that lines 712 and 153 expressed about 50% less β-arrestin-1 and -2 than the WT parental line expressing scrambled shRNA sequences. (B) Unsupervised heirarchical clustering showing alterations of gene expression in the GT1-7 lines (712 and 153) relative to wild type. ANOVA was used to determine those probesets changing significantly (*P*<0.05) at least 1.5 fold in 712 and/or 153 versus WT. This list was then subjected to heirarchical clustering using the average linkage algorithm and displayed as a heat map (representative samples shown). Red: high expressor, blue: low expressor. Color scale: log(BASE 2) normalized intensity.

Thus far, our studies utilized β-arrestin KO MEFs expressing exogenous GPR54 to determine the roles of β-arrestin in GPR54 signaling. To complement this study, we also examined the roles of β-arrestin in regulating gene expression in GT1-7, a hypothalamic neuronal cell line that expresses GPR54 endogenously [26]. GT1-7 cell lines stably expressing shRNAs against β-arrestin-1 (line 712) and β-arrestin-2 (line 153) were created. Western blotting data revealed that lines 712 and 153 expressed about 50% less β-arrestin-1 and -2 than the WT parental line expressing scrambled shRNA sequences (Fig. 9A). WT and β-arrestin-1 and -2 downregulated GT1-7 cells were serum-starved overnight and then treated with Kp-10 for 6 hours in serum-free media. Total RNA was extracted and used to conduct a microarray analysis of gene expression using the Affymetrix Mouse Gene 1.0 ST gene array. Our analysis revealed that relative to WT GT1-7 expressing scrambled shRNA sequences, the β-arrestin-1 and -2 downregulated lines displayed a significantly (*P*<0.05) different pattern of gene expression following Kp-10 treatment (Fig. 8B). We also found that reduced β-arrestin-1 and -2 levels resulted in gene expression patterns that were higher, lower or unchanged relative to each other (Fig. 9B).

## Discussion

The neuropeptide Kp and its cognate receptor, GPR54 are major regulators of the HPG axis and loss-of-function mutations in GPR54 were found to cause an absence of puberty and hypogonadotropic hypogonadism [2], [3]. Despite the physiological and clinical importance of GPR54, to date only a very small number of studies have revealed how this receptor signals intracellularly. Until our recent study where we demonstrated that GPR54 regulates ERK activity in a β-arrestin-dependent manner in the invasive breast cancer cell line, MDA MB-231 [4], it was largely assumed that GPR54-regulated cell functions occurred via the G_q/11_ pathway. Having determined that this was not the case, we wanted to understand in-depth the contributions of the GPR54-coupled G_q/11_ and β-arrestin pathways in regulating cell function following Kp treatment. To do this, we chose ERK activation as our biological readout and employed the use of MEFs derived from the β-arrestin-1, -2 (single) and -1/2 (double) KO mice as well as the G_q/11_ (double) KO mice [27], [28]. Cells derived from these animals have been used extensively to study the roles of various β-arrestin- and G_q/11_dependent events downstream of multiple GPCRs. For example, the β-arrestin KO MEFs have been used to study the differential effects of β-arrestins on the internalization, desensitization and activation of ERK downstream of protease activated receptor-2 [29] and the roles of β-arrestins in the lysophosphatidic acid-induced NF-κB activation [30]. The G_q/11_ double KO MEFs have been used to study the bradykinin-mediated MAPK activation [31] and the regulation of PLCβ1a membrane anchoring by its substrate phosphatidylinositol (4,5)-bisphosphate [32].

We began our study by determining the effect of Kp-10 concentration on ERK activation and found that identical to what Kotani et al. [1] observed for human GPR54 expressed in CHO-K1 cells, as little as 10 nM Kp-10 could trigger ERK activation in the β-arrestin-1 and -2 WT MEFs with a saturable effect being achieved with 100 nM Kp-10. Next we demonstrated that in the WT MEFs, 100 nM Kp-10 triggered an early (within 5 minutes) and robust response in ERK activation that was greatly diminished within 30 minutes. The rapid activation in ERK was similar to that described in CHO-K1 cells stably expressing GPR54 [1]; in COS-7 cells transiently expressing GPR54 [24] and in GT1-7 mouse hypothalamic neurons expressing endogenous GPR54 [31]. The diminished response in ERK activation in the WT MEFs was almost identical to that observed in the GT1-7 neurons [33] and similar to that seen in the COS-7 cells [24]. However, unlike what we observed in the WT MEFS, Kotani et al. (2001) [1] continued to observe very high levels of Kp-10-dependent ERK activity after 30 minutes in CHO-K1 cells. These results therefore reveal that GPR54 is functionally coupled to ERK activation in MEFs and the characteristics of this activation are similar to that observed in other cellular systems expressing both endogenous and exogenous GPR54.

The β-arrestin and G_q/11_ KO MEFs have proven to be highly effective cell models for studying the role of β-arrestin and G_q/11_ in the activation of MAPK by various GPCRs [19]; [29], [31]. Here, based on our findings, we also demonstrate that these cell lines are a robust model for studying the contributions of the GPR54-coupled G_q/11_ and β-arrestin pathways in mediating ERK activation. Once this was established, we began by determining what effect a loss of β-arrestin expression would have on GPR54-dependent ERK activation in MEFs. Our data showed that a loss of both β-arrestin-1 and -2 triggered a reciprocal effect on ERK activation (β-arrestin-2 potentiates and β-arrestin-1 inhibits). This finding is consistent with findings previously reported for the AT1AR in HEK-293 cells [9] and suggested for the V2R [20]. A reciprocal effect has also been documented for PAR1, however, here β-arrestin-2 inhibits and β-arrestin-1 potentiates ERK activation [34]. Reciprocal regulation is not mechanistically understood, nor does it apply to all 7TMRs. For example, the β2AR and PTH1R exhibit a marked reduction in ERK activation following siRNA depletion of either β-arrestin1 or -2 [10], [13].

Our assessment of the role of the G_q/11_ pathway in the GPR54-dependent activation of ERK reveals that GPR54 simultaneously requires G_q/11_ and β-arrestin-2, as early as 2.5 minutes following Kp-10 treatment, to trigger maximum ERK activation. Based on RNAi studies examining ERK activation downstream of the AT1AR, β2AR, V2R and PTH1R in HEK 293 cells [9], [10], [13], [20], the G_q/11_ and β-arrestin-mediated ERK pathways are temporally distinct and independent of each other. However, for the chemokine receptor CCR7, CXCR4, lysophosphatidic acid receptor and the AT1AR, β-arrestin-dependent signaling was demonstrated to be dependent on Gi signaling since pertussis toxin treatment eliminated or reduced the β-arrestin-dependent signaling events [33]–[37]. Due to the design of these studies the temporal aspects of the G_q/11_ and β-arrestin-mediated signaling events could not be addressed. In 2005, however, Barnes et al. [22] demonstrated that the AT1AR-dependent activation of RhoA requires the concurrent cooperation of the G_q/11_ and β-arrestin-1-dependent pathways. In this study they observed that neither pathway alone was sufficient to robustly activate RhoA, however, the concurrent recruitment of β-arrestin-1 and activation of G_q/11_ led to full activation of RhoA and to the subsequent formation of stress fibers. This led them to conclude that both pathways were co-dependent with respect to RhoA activation.

Finally, both the immunoblot and immunofluorescence analyses of pERK levels in the cell following Kp-10 treatment were in general agreement with each other and when considered together, the biochemical and immunofluorescence data strongly suggest that downstream of GPR54, β-arrestin-1 negatively regulates the nuclear accumulation of pERK while β-arrestin-2 and G_q/11_ are simultaneously required for pERK nuclear localization. Therefore, both G_q/11_ and β-arrestins presumably regulate ERK-dependent gene transcription by directly regulating the nuclear level of pERK.

Based on our data, we also conclude that GPR54 couples to the G_q/11_ and β-arrestin pathways in a co-dependent and temporally overlapping manner to regulate ERK activity. While this co-dependence is significant, it is not absolute, since loss of either G_q/11_ or β-arrestin-2 results in a greatly diminished but not a complete loss of ERK activation. Recently, we reported that GPR54 couples to β-arrestin-2 via sequences in its second intracellular loop [4] and another study reported that the L148 residue in the second intracellular loop of GPR54 is required for the activation of G_q/11_ [25]. Taken together, it is possible that G_q/11_ and β-arrestin-2 might be interacting with each other on the second intracellular loop and this interaction might account for the observed co-dependence. With respect to the temporal overlap, for many 7TMRs, β-arrestin only interacts with the receptor following receptor/G protein interaction. However, we recently reported that GPR54 is constitutively associated with β-arrestin-1 and -2 [4], thus the temporal overlap between the GPR54-coupled G_q/11_ and both β-arrestin pathways is consistent with this observation.

To date, a number of naturally occurring GPR54 “loss-of-function” mutants have been identified [38]. These mutants are associated with the condition of hypogonadotropic hypogonadism (HH) in humans and have also been demonstrated to be uncoupled from the G_q/11_ pathway, as evidenced by their inability to stimulate inositol phosphate formation [38]. We recently determined that the GPR54 mutant, R331X, is uncoupled from this pathway because it is not expressed at the plasma membrane [4]. Using L148S, a functionally G_q/11_uncoupled GPR54 mutant that is still expressed at the plasma membrane [25], we demonstrated it could still interact with β-arrestins, a strong indication it and other such mutants are capable of signaling independently of G_q/11_. Next, we demonstrated that even when uncoupled from G_q/11_, GPR54 continues to strongly modulate the expression of many genes. Taken together, our data implies that while many naturally occurring GPR54 mutants are uncoupled from the G_q/11_ pathway, they are only “loss-of-function” mutants with respect to this pathway. This finding, if recapitulated in physiologically relevant cells, will be potentially significant for both the mutant and WT receptor. For the mutant receptor it begs the question as to what roles this receptor continues to play in the HH individual. For the WT receptor it opens up the possibility of developing ligand-directed signaling therapies to promote or reduce certain receptor mediated events, under both healthy and pathophysiological conditions as has been demonstrated for the β-adrenergic receptor [39].

Most of our conclusions in this study are based on findings made in KO MEFs expressing exogenous levels of GPR54. It therefore remains possible that our results, though fully valid, are specific to these cells only. Since GPR54 is expressed on GnRH neurons in the hypothalamus and is best understood as a regulator of hypothalamic GnRH release [3], we tested the hypothesis, based on data derived from the KO MEFs that diminished β-arrestin 1 and 2 signaling in the GnRH neuron results in altered downstream responses, such as gene expression. If this was the case it would confirm a role for β-arrestin in regulating GPR54 signaling. We tested our hypothesis in the GT1-7 cell system. GT1-7 is a neuronal cell line derived from the mouse hypothalamus; it expresses GPR54 endogenously and is an established neuronal model for studying GPR54 signaling in the hypothalamus, specifically as it relates to triggering GnRH release [26]. As predicted, our data revealed that a loss of β-arrestin-1 and -2 expression in GT1-7 triggered significant and distinct patterns of responses (increased, decreased and no change) in gene expression following Kp-10 treatment, thereby confirming β-arrestin-1 and -2 are major mediators of GPR54 signaling. Recently another study also highlighted possible differential roles for β-arrestin-1 and -2 in regulating GPCR function in neurons. In a recent study conducted in hippocampal neurons, Lelouvier *et al.* [42] demonstrated by live-cell imaging that following the activation of the somatostatin type 2A receptor (SST2A), β-arrestin-1 and -2 were recruited to the plasma membrane but that β-arrestin-1 also translocated to the nucleus, suggesting that this protein could serve as a “nuclear messenger” for the SST2A receptor in hippocampal neurons.

GPR54 is a 7TMR of tremendous physiological and clinical importance. Although, first identified in 1999 as an orphan receptor [40], the signaling potential of this receptor is gradually being uncovered. In 2009, our group was the first to demonstrate that GPR54 undergoes GRK-dependent desensitization; can recruit and interact with β-arrestin and activate ERK in a β-arrestin-dependent manner [4]. This finding was significant since it opened up the possibility that some GPR54-regulated functions are β-arrestin-dependent. The present study now very convincingly confirms this. Overall, our studies highlight the importance of β-arrestin in regulating 7TMR signaling in MEFs, GT1-7 hypothalamic neurons and as we previously reported in MDA MB-231 human breast cancer cells [4]. Thus it appears that the GPR54 signaling mechanisms uncovered in this study might be conserved in various cellular systems.

## Materials and Methods

### Materials

Restriction enzymes were obtained from New England Biolabs Inc. (Pickering, ON, Canada). Kisspeptin-10 was purchased from Phoenix Pharmaceuticals (Burlingame, CA). Rabbit monoclonal anti-ERK1/2 and anti-phospho ERK1/2 antibodies were from Cell Signaling Technology (Pickering, ON, Canada). Mouse monoclonal anti-DDK (FLAG) antibody was obtained from Origene (Rockville, MD). Mouse monoclonal β-arrestin-1 and -2 antibodies were obtained from Upstate Biotechnology (Lake Placid). Rabbit polyclonal anti-Gq antibody was from Santa Cruz Biotechnology (Santa Cruz, CA). Alexa Fluor 568-conjugated anti-rabbit IgG secondary antibody and Hoechst dye were acquired from Invitrogen (Burlington, ON, Canada). Fetal bovine serum and all other cell culture reagents were purchased from Invitrogen (Burlington, ON, Canada). All other reagents were purchased from BioShop, Fisher Scientific, VWR, GE and Corning.

### Cell Lines

Mouse embryonic fibroblast (MEF) cell lines derived from the β-arrestin-1 and β-arrestin-2 single and β-arrestin-1/2 double knockout mice were developed in the laboratory of Dr. Robert Lefkowitz [27]. The β-arrestin-2 single and β-arrestin-1/2 double knockout mice were derived from the same WT strain and accordingly, the same WT MEFs (referred to as WT2) serve as their WT parents in this study. MEF G_q/11_ knockout and wild-type cell lines were developed in the laboratory of Dr. Stefan Offermanns and previously described in [28]. The GT1-7 cells were obtained from Drs. Denise Belsham (University of Toronto) and Pamela Mellon (University of California, San Diego) [41].

### Plasmid Constructs

A 1607-bp cDNA clone encoding human GPR54 was purchased from Origene (NM_032551.3) and used as a template to amplify the 1197-bp ORF by PCR. The FLAG-epitope, engineered into 5′ primer sequences, was introduced at the amino terminus of GPR54 by PCR. FLAG-GPR54 was then cloned into the NheI and NotI sites of a homemade mammalian expression vector derived from the pEGFP-C vector backbone (Invitrogen). The 1197-bp ORF was also cloned into the BglII and HindIII sites of pEYFP-N1. For the ‘add-back’ experiments, β-arrestin-1-GFP and β-arrestin-2-GFP were used. The nature of these constructs was previously described in Pampillo et al., 2009. The Gα_q_ cDNA (human) was obtained from Dr. Stefan Offermanns.

### Cell Culture and Electroporation

MEF β-arrestin-1 knockout, β-arrestin-2 knockout, β-arrestin-1/2 double knockout, G_q/11_ knockout and the corresponding wild type parental cell lines were all maintained in DMEM supplemented with 10% FBS, 1% penicillin/streptomycin (v/v) and 1% (v/v) non-essential amino acids. GT1-7 cells were grown in monolayer in DMEM supplemented with 10% FBS, 4.5 mg/ml glucose and 1% penicillin/streptomycin (v/v). All cell lines were maintained at 37°C in a humidified atmosphere containing 5% CO_2_. When confluent, all MEF cell lines were transiently transfected with either 20 µg of FLAG-tagged GPR54 (FLAG-GPR54) or EYFP-tagged GPR54 (GPR54-EYFP) cDNA by electroporation using the Bio-Rad Gene Pulser Xcell System (exponential decay protocol: 230 V, 950 µF) with BioRad 0.4 cm electroporation cuvettes. For the β-arrestin and G_q_ ‘add-back’ experiments, the appropriate MEF knockout cell lines were co-transfected with 15 µg of FLAG-GPR54 and 20 µg of either β-arrestin1-GFP, β-arrestin2-GFP, G_q_, or GFP vector (control) cDNA using the same electroporation protocol described above. Following electroporation (16–18 hours), cells were split to 6-well plates (ERK1/2 activation assays) or 18 mm glass coverslips in 12 well plates (immunostaining). The cells were then allowed to recover for approximately 6 hours prior to overnight serum starvation in serum-free media.

### Generation of β-arrestin-1 and -2 shRNA downregulated GT1-7 (mouse) cell lines

GT1-7 neurons were transfected with shRNAs (OriGene Technologies) against β-arrestin 1 (sequence cloned in the pGFP-V-RS Vector: GACTCCAGTAGACACCAATCTCATAGAGC) and β-arrestin 2 (sequence cloned in the pGFP-V-RS Vector: GTGGCTCAGCTAGAACAAGATGACCAGGT) using Lipofectamine (Invitrogen) to create the stable cell lines GT1-7 712 and 153, respectively. Heterogeneous populations of stable transfectants were selected in media containing 0.5 µg/mL of puromycin and maintained on media containing 0.25 µg/mL of puromycin. β-arrestin knockdown was confirmed by western blot analysis.

### Western Blot Analysis of ERK1/2 Phosphorylation

MEF knockout (β-arrestin-1KO, -2KO, and -1/2 KO; G_q/11_ KO) and wild-type cell lines overexpressing FLAG-GPR54 were used in ERK1/2 activation assays. Prior to experimentation, overnight serum-starved wild-type or knockout MEF cells were placed in Hanks' balanced salt solution (HBSS: 1.2 mM KH2PO4, 5 mM NaHCO3, 20 mM HEPES, 11 mM glucose, 116 mM NaCl, 4.7 mM KCl, 1.2 mM MgSO4, 2.5 mM CaCl2, pH 7.4) for 30 minutes. Assays were then conducted by treating these cells with Kp-10 for the indicated times (see figures). Following stimulation, cells were placed on ice then solubilized in lysis buffer (25 mM HEPES, pH 7.5, 300 mM NaCl, 1.5 mM MgCl_2_, 0.2 mM EDTA, 0.1% Triton X-100) containing both protease inhibitors (AEBSF, leupeptin, aprotinin) and phosphatase inhibitors (sodium fluoride, sodium orthovanadate), and then clarified by centrifugation for 20 minutes at 23 000 RCF. 50 µg of protein was subjected to SDS-PAGE and subsequently transferred to nitrocellulose membranes for immunoblotting. Immunoblots were then analyzed for phosphorylated ERK1/2 using a phosphospecific rabbit monoclonal antibody (1∶2,000) (Phospho p44/42 MAPK, Thr202/Tyr204) from Cell Signaling Technology and for total ERK1/2 using a rabbit monoclonal antibody (1∶2,000) (p44/42 MAPK) from Cell Signaling Technology. Chemiluminescent detection was performed using an anti-rabbit horseradish peroxidase-conjugated secondary antibody (1∶2500) and developed with ECL (GE Healthcare, Piscataway, NJ). Immunoblots were quantified by densitometry using the 4.0.2 version of the data acquisition and analysis software from Scion Corp. (Frederick, MD).

### Co-immunoprecipitation

Transiently transfected (FLAG-L148S and β-arrestin 1-GFP or β-arrestin 2-GFP) HEK 293 cells were serum starved for 30 mins in HBSS at 37°C and then stimulated with 100 nM Kp-10 for 5 mins. Cells were then solubilized in lysis buffer containing protease inhibitors. FLAG-L148S was immunoprecipitated from 750 µg of total protein with FLAG-agarose beads (Sigma). The proteins were analyzed by SDS-PAGE and western blotting as described in the figure legends. Immunoblots were visualized by chemiluminescence using an ECL kit.

### Confocal Microscopy and Immunostaining

Transiently transfected MEF knockout and wild-type cells overexpressing GPR54-EYFP were used in the ERK1/2 localization studies. Following overnight serum starvation, all cells were placed in HBSS for 30 minutes and then treated with Kp-10 or HBSS (vehicle) for 10 minutes. Cells were fixed and permeabilized using 4% formaldehyde and 0.2% Triton-X in HBSS for 20 minutes before the addition of primary antibody. Cells were incubated with the phosphospecific ERK1/2 rabbit monoclonal antibody (1∶300) (Phospho p44/42 MAPK, Thr202/Tyr204) overnight at 4°C, followed by incubation with secondary antibody (goat anti-rabbit antibody conjugated to AlexaFluor 568 was used at 1∶1200) for 45 minutes at room temperature. Cells were then counter-stained using Hoechst at 1∶50000 (v/v) for 7 minutes at room temperature to detect nuclei. All coverslips were mounted in IMMU-MOUNT (Thermo Shandon, Pittsburgh, PA) onto glass slides and allowed to air dry before viewing. Confocal analysis was performed on an Olympus Fluoview 1000 laser scanning confocal microscope using the 40 X Plan Superapochromat 0.9 and the 60X Plan Apochromat 1.42 oil objective. Confocal imaging studies were performed using multiple excitation (405, 515 and 559) and emission (band pass 425–475 nm, 530–630 nm and 575–675 nm for Hoechst, EYFP and AlexaFluor 568 respectively) filter sets. Multi-colour images were acquired in the sequential acquisition mode to avoid cross-excitation. During image acquisition, the same image acquisition settings were applied, thus direct comparisons could be made between cell lines.

### Gene Expression Microarray Analysis

G_q/11_ KO MEFs and their WT parents as well as the β-arrestin-1 and -2 downregulated GT1-7 lines (712 and 153) and their WT control GT1-7 cells were serum-starved overnight followed by a wash in HBSS and a second incubation in serum-free media for one hour. Cells were then treated with Kp-10 for six hours in serum-free media, following which total RNA was isolated using the RNeasy Mini Kit from Qiagen (Missassauga, ON, Canada) according to the manufacturer's instructions. Total RNA was subjected to an on column DNase digest to eliminate any possible genomic DNA contamination using the recommended on column RNase-free DNase kit (Qiagen). Total RNA was then subjected to microarray analysis at the London Regional Genomics Centre (London, ON, Canada). RNA was biotinylated and hybridized to Mouse 1.0 GST Gene Array (Affymetrix, Santa Clara, CA). Array washing, scanning and probe quantification were carried out as per the manufacturer's instructions using GCOS software [43] except that the target intensity was set to 150. For each array, GCOS output was imported as CEL files into Partek Genomic Suite software (Partek, St. Louis MO), and data were normalized using the RMA (Robust Multichip Averaging) algorithm. ANOVA with nominal alpha value set to 0.05 was then used to determine those probe sets significantly different between the test and control samples.

### Data Analysis

The means ± S.E are shown for values obtained for the number of independent experiments indicated in the figure legends. GraphPad Prism software (Graph Pad, San Diego, CA) was used to analyze data for statistical significance, as well as to analyze and fit dose-response data. The statistical significance was determined by one-way analysis of variance with Dunnett's multiple comparison post hoc test and two-tailed *t* test.
